# Identification and Characterization of Novel Fc-Binding Heptapeptides from Experiments and Simulations

**DOI:** 10.3390/polym10070778

**Published:** 2018-07-16

**Authors:** Xiaoquan Sun, Justin Weaver, Sumith Ranil Wickramasinghe, Xianghong Qian

**Affiliations:** 1 Department of Biomedical Engineering, University of Arkansas, Fayetteville, AR 72701, USA; xs005@uark.edu; 2 Department of Chemical and Biological Engineering, Colorado State University, Fort Collins, CO 80523, USA; justinrossweaver@gmail.com (J.W.); swickram@uark.edu (S.R.W.); 3 Ralph E Martin Department of Chemical Engineering, University of Arkansas, Fayetteville, AR 72701, USA

**Keywords:** protein A, heptapeptide, M13 phage display, ELISA, Fc, purification, docking, molecular dynamics simulations

## Abstract

Purification of biologically-derived therapeutics is a major cost contributor to the production of this rapidly growing class of pharmaceuticals. Monoclonal antibodies comprise a large percentage of these products, therefore new antibody purification tools are needed. Small peptides, as opposed to traditional antibody affinity ligands such as Protein A, may have advantages in stability and production costs. Multiple heptapeptides that demonstrate Fc binding behavior that have been identified from a combinatorial peptide library using M13 phage display are presented herein. Seven unique peptide sequences of diverse hydrophobicity and charge were identified. All seven peptides showed strong binding to the four major human IgG isotypes, human IgM, as well as binding to canine, rat, and mouse IgG. These seven peptides were also shown to bind human IgG4 from DMEM cell culture media with 5% FCS and 5 g/L ovalbumin present. These peptides may be useful as surface ligands for antibody detection and purification purposes. Molecular docking and classical molecular dynamics (MD) simulations were conducted to elucidate the mechanisms and energetics for the binding of these peptides to the Fc region. The binding site was found to be located between the two glycan chains inside the Fc fragment. Both hydrogen bonding and hydrophobic interactions were found to be crucial for the binding interactions. Excellent agreement for the binding strength was obtained between experimental results and simulations.

## 1. Introduction

Pharmaceutical sales, and in particular biologically derived pharmaceutical sales, are increasing [1]. In fact, many of the top selling pharmaceuticals are monoclonal antibodies (mAb). However, biologically derived pharmaceuticals—including mAbs—are much more expensive than typical small-molecule therapeutics due to the complexity of both the molecule and the production process. Downstream purification of these mAbs can account for almost 80% of the total cost of manufacturing suggesting opportunities for novel antibody purification technology [2]. Thus, there is a continual need to develop new, more efficient purification processes. Recently, much work has gone into finding possible functional replacements for protein A which is the workhorse affinity technology for mAb purification. Drawbacks of protein A include the fact that it is expensive and unstable under typical column cleaning/sanitization conditions such as 1M NaOH [3,4,5].

Derivatives of protein A have been shown to retain similar binding properties while showing an increase either in binding capacity or stability [6,7]. A few alkaline-stabilized protein A derivatives are currently marketed as chromatography resins such as the GE Healthcare (Pittsburgh, PA, USA) MabSelect Sure resin which uses a modified tetrameric B binding domain, Protein A Ceramic HyperD F resin from Pall Corporation (Port Washington, NY, USA) and Tosoh Biosciences (South San Francisco, CA, USA) Toyopearl AF-protein A-650F resin which uses a tetrameric derivative of the C binding domain of protein A. However, protein A derivatives still suffer from high costs associated with licensing fees and costs of producing the recombinant protein. 

Therefore, synthetic ligands that have little or no protein A homology have received a good deal of academic and industrial interest over the past decade. Totally synthetic chemical ligands, such as the multi-modal hydrophobic charge induction MEP Hypercel^®^ ligand from Pall Corporation which displays both charge and hydrophobic binding characteristics, are actively being investigated. Wang et al. showed that by screening parallel small libraries of combinatorial chemical ligands certain ligands could perform chromatographic separation of BSA and avidin [8]. Haigh et al. identified small molecule immunoaffinity ligands based on a multicomponent Ugi reaction combinatorial library [9]. A very interesting study in 2011 by Arnold et al. demonstrated a method for evaluating large libraries of small molecules for Fc binding based on surface plasmon resonance (SPR) analysis of chemical microarrays [10]. Several chemical ligands were identified with potential Fc binding properties. While these chemical-based ligands show promise as yet. They have generally not shown comparable selectivity to protein A.

Synthetic peptides are also currently being explored as possible antibody purification ligands [11]. Small peptides have several advantages over both large protein A derivatives and small-molecule chemical affinity ligands. Due to the much smaller size and reduced complexity of peptides as compared to large protein A derivatives, peptides may be inherently more stable. Short peptides do not assume complex tertiary structures like that found in protein A but may or may not form helical secondary structure [12]. Secondary structures such as α-helices and β-sheets are thermodynamically favored. Furthermore, recovery of these structures after denaturation is generally rapid and much more reproducible than recovery of tertiary structures. Therefore, recovery of active peptide after exposure to unfavorable folding conditions is more likely than recovery of large proteins after similar exposure. 

There have been multiple peptide-based ligands identified as Fc binders or specific protein binders using various selection methods [13,14,15,16,17,18,19]. The unusual peptide PAM, first described by Fassina et al. in 1996, has very promising antibody purification characteristics, producing antibody with greater than 90% purity from clarified cell supernatant. [20,21]. Another peptide, HWRGWV, was identified by Yang et al. via an elegant radiolabeled target screening method of a solid phase hexapeptide resin library [22]. The HWRGWV peptide has been shown to demonstrate good performance for purification of hIgG from CHO cell culture media, and Fc binding site data has been obtained [23,24]. Impurity removal was also studied, showing good removal of DNA and host cell proteins. A common method used to identify promising peptides is panning of peptides from phage display libraries. This is the method used in this work.

Phage display is a method for evaluating a very large protein library for specific sequences that bind the desired target. Today, the method is used to study molecular biology mechanisms involving protein–protein and protein–non-protein interactions [4,25,26,27,28,29,30]. Protein libraries of varying length, from short peptides to full antibody, are “displayed” on the surface of bacteriophage coat proteins. Since the protein displayed on the surface is coded by the genetic material within the viral genome there is a direct link between the phenotype of the phage and its genotype, thereby allowing DNA sequencing and identification of the displayed protein. M13 phage is the most common phage currently used, and is the one used in this study in the form of the Ph.D-7 library from New England BioLabs Inc. (Ipswich, MA, USA) [31,32].

In this contribution, seven novel heptapeptides have been identified through a series of solution affinity panning experiments that have specific affinity for the Fc region of immunoglobulins. To our knowledge, there have not been any previously reported heptapeptide Fc binding ligands in the public literature. Importantly, these peptides may be excellent candidates for either antibody detection or purification ligands once immobilized. To that end, binding experiments were conducted via ELISA with hIgG4 coated wells in challenging cell culture media (DMEM with 5% FCS and 5 g/L BSA) to demonstrate specificity. Protein A has known binding tropisms for hIgG1, hIgG2, and hIgG4 but is a poor hIgG3 binder [33]. Therefore, binding to various human and non-human antibodies has also been explored.

In order to understand the mechanisms of binding for the identified seven novel heptapetides, theoretical studies including molecular docking and classical molecular dynamics (MD) simulations were conducted. A consensus binding site (CBS) located at the hinge of the Fc region in antibodies was discovered by X-ray crystallography [34]. CBS is known as the binding domain of Protein A [35], Protein G [36], as well as for some other proteins or peptides [37,38,39]. Earlier classical MD simulations [38,40] have been performed to investigate this binding site. However, the exact binding mechanisms between the synthetic ligands and antibodies remain unclear due to the lack of crystal structures of the antibody-ligand complexes formed. Earlier studies using molecular docking and MD simulations found that synthetic ligand MEP also binds to the CBS region [41,42]. However, the docking studies conducted only used one Fc fragment chain without the glycan chains in the CBS domain. Other studies of synthetic ligands did not take into account these glycan chains [43,44,45,46] either. Molecular docking is a reliable method and has been well validated in many cases with structures extracted from X-ray crystallography [47,48,49,50,51,52]. The key to obtaining accurate results is to start with the correct assumptions and structures. Here the two glycan chains were taken into account during our docking studies. The two glycan chains are located at the center of the Fc fragment connected via the *N*-glycan linkage at the two ASN residues forming a highly polar and hydrophilic region. The compositions of the two chains are similar—including *N*-acetylglucosamine, mannose, fucose, and galactose. Chain 1 has one more fucose molecule than Chain 2. Glycan chains are known to play an important role in the functions of antibodies [53,54]. However, the role of glycan chains in the binding interactions between the antibodies and ligands remains largely unknown.

Protein A binds to the Fc fragment through residues at the CBS region and stays outside the Fc fragment without interactions with glycan chains located inside the Fc fragment. However, small synthetic ligands are more flexible in conformation and can penetrate via the gap between the two Fc chains to reach the glycan chains. In this study, the whole Fc fragment of hIgG4 was used as the model target protein including the two heavy chains and two glycan chains, and seven ligands identified experimentally. The molecular interactions between ligands and the Fc fragment were analyzed using molecular docking and MD simulations. A rigid docking approach with AutoDock was first carried out to search for the possible binding sites on the Fc fragment, followed by the flexible docking approach to optimize the binding of the ligand and to evaluate the binding free energy. One Fc-ligand complex with the strongest binding affinity was selected for MD simulations. The interaction energies between Fc fragment and the ligand were analyzed. The contributions of the amino acids on the ligands to the binding interaction were also investigated. This study would shed light on the specificity of IgG-ligand interaction and help design new ligands with high affinity to IgG.

## 2. Materials and Methods

### 2.1. Reagents

Ph.D.-7 phage-peptide library and F^+^ *E. coli* strain K12 ER2738 were purchased from New England BioLabs (Ipswich, MA, USA). 2xYT media (powder mix, 31 g/L) was purchased from Amresco (Solon, OH, USA). Tetracycline hydrochloride (antibiotic), bovine serum albumin (BSA), lactoferrin, and horseradish peroxidase enzyme chromogenic substrate ABTS (2,2′-Azino-bis(3-ethylbenzthiazoline-6-sulfonic acid) diammonium salt) was obtained from Sigma-Aldrich Corp. (St. Louis, MO, USA). High glucose DMEM media and fetal calf serum (FCS) were obtained from HyClone, a division of Thermo Fisher Scientific (Waltham, MA, USA). Human IgG4 was obtained through a generous donation by Eli Lilly (Indianapolis, IN, USA). Human IgG1, IgG2, IgG3, and IgM, canine IgG, murine IgG, and rat IgG were purchased from Athens Research & Technology, Inc. (Athens, GA, USA). Microlon 200 medium-binding, clear, flat-bottomed, polystyrene 96-well plates were purchased from USA Scientific (Ocala, FL, USA). All buffer reagents including mono- and di-basic phosphate, Tris base and Tris hydrochloride, L-glycine, hydrochloric acid, cysteine HCl, EDTA disodium salt, Tween 20, citric acid, glacial acetic acid, sodium hydroxide, glycine, 30% hydrogen peroxide, and NaCl were purchased from JT Baker (Philipsburg, NJ, USA). A Biomax PES 5 kDa MWCO TFF membrane was purchased from EMD Millipore (Billerica, MA, USA). Papain proteolytic enzyme, protein A, streptavidin agarose beads, and EZ-Link Sulfo-NHS-Biotin no weigh format were all purchased from Pierce Biotechnology, Inc. (Rockford, IL, USA). Isopropyl β-d-1-thiogalactopyranoside (IPTG) and 5-bromo-4-chloro-indolyl-β-d-galactopyranoside (X-Gal) were purchased from Fermentas, Inc. (Glen Burnie, MD, USA). Affi-Gel 10 activated immunoaffinity agarose resin was obtained from Bio-Rad (Hercules, CA, USA). Anti-M13 HRP-conjugated antibody was purchased from GE Healthcare (Piscataway, NJ, USA).

### 2.2. Fc Target Preparation

#### 2.2.1. Fc Generation

Human IgG4 (hIgG4) monoclonal antibody was digested with papain that had been immobilized on Affi-Gel 10 agarose beads and then purified over a protein A conjugated agarose column to obtain enriched Fc. Originally in citrate buffer pH 6.7, hIgG4 was diafiltered using 10 diavolumes into 20 mM phosphate, 150 mM NaCl, 10 mM EDTA, pH 7.0 (PBES), using a 5 kDa MWCO Biomax TFF membrane module and then concentrated to 20 mg/mL. Papain (5 mg lyophilized) was reconstituted with 1 mL of 20 mM phosphate containing 150 mM NaCl at pH 7.0 (PBS). Next 0.2 mL of 50% slurry of Affi-Gel 10 was transferred to a 1.7 mL centrifuge tube, washed with cold DI water, and equilibrated in PBS according to the product instructions. 

The 0.2 mL of equilibrated 50% Affi-Gel 10 slurry (0.1 mL total bead volume) was added to the 1 mL of 5 mg/mL papain solution in a centrifuge tube and allowed to conjugate for 4 h with agitation at 4 °C. The effectiveness of conjugation was determined by measuring the absorbance at 280 nm of the reaction supernatant (diluted 1:10 in 0.1 M HCl). Any unreacted sites on the Affi-Gel 10 were quenched by incubation with 0.1 M glycine overnight at 4 °C with agitation. Then 0.1 mL of the immobilized papain was activated prior to digestion for 30 min at 37 °C with 1 mL PBES containing 10 mM freshly added cysteine (pH was readjusted to 7.0). The Affi-Gel 10 resin with immobilized papain was pelleted by centrifugation. 

The resin was resuspended in 1 mL of hIgG4 solution (prepared by adding 0.5 mL PBES containing 20 mM cysteine to 0.5 mL of 20 mg/mL hIgG4 for final concentration of 10 mg/mL) and allowed to digest at 37 °C for six hours with agitation. The immobilized papain containing digested hIgG4 mixture was centrifuged to pellet the resin and the supernatant containing hIgG4 fragments was collected, diluted with 9 mL PBS and immediately loaded over 1 mL Affi-Gel Protein A conjugated resin packed in a 5/100 tricorn column (GE Healthcare Pittsburgh, PA, USA) on an AKTA FPLC (GE Healthcare Pittsburgh, PA, USA). 

Protein A (5 mg lyophilized) was reconstituted and conjugated to 1 mL Affi-Gel 10 resin following the same procedure for papain conjugation. The immobilized protein A was equilibrated with PBS prior to loading. The column containing resin with immobilized portion A was operated at 0.5 mL/min throughout the run. After loading, the column was washed with 10 column volumes of PBS and the bound material eluted with 0.1 M acetic acid and neutralized to pH 7.0 with 1 M phosphate pH 8.5. 

Papain digestion of the antibody in the presence of a mild reducing agent generates Fc and a mixture of Fab and fragments. Protein A resin binds to the Fc fragments strongly while Fab predominantly passes through the column unbound; therefore, the eluted fraction is highly enriched in Fc fragments as well as any undigested antibody. Fc enrichment after protein A purification was observed on a non-reducing 12% SDS-PAGE gel.

#### 2.2.2. Fc Biotinylation

1 mL of 0.1 mg/mL Fc in acetate/phosphate (as described in Section 2.2.1) buffer pH 7.0 was biotinylated using the EZ-Link Sulfo-NHS-Biotin no weigh format at the recommended conditions. Briefly we aimed for a 20-fold excess of sulfo-NHS-biotin reagent. Therefore, 4 µL of 10 mM sulfo-NHS-biotin was added to 1 mL of 0.1 mg/mL Fc (~50 kDa) solution and incubated for 2 h on ice. Biotinylated Fc was diluted with 10 mL (of 50 mM Tris containing 150 mM NaCl at pH 7.5 (TBS buffer) and concentrated to 0.1 mg/mL (1 mL) using an EMD Millipore Amicon Ultra-15 5 kDa MWCO spin concentrator in a swing-bucket rotor centrifuge at 3000 rpm for approximately 40 min. 

### 2.3. Phage Display Panning

The Ph.D.-7 heptapeptide phage library was supplied at 2 × 10^13^ pfu/mL and had a sequence diversity of approximately 2 × 10^9^ unique codon sequences. The panning experiments generally followed the procedure outlined in the Ph.D. phage display library manual. A 100-fold representation of the sequence diversity, 2 × 10^11^ phage particles, was added to the first round panning reaction. Initially, several panning experiments were conducted with Fc (non-biotinylated) being directly adsorbed to the surface of polystyrene dishes. Though these panning experiments yielded clear consensus sequences after three rounds of panning, it was subsequently discovered that all clones showed appreciable plastic binding and not Fc binding, even with strenuous (1% Tween 20) surfactant washes. Such behavior has been detected before [25,55]. Therefore a solution panning system was employed with no polystyrene present. In the first round of panning 2 × 10^11^ library phage particles were diluted in 200 µL TBS with 0.1% Tween 20 and 2 pmol biotinylated Fc (100 ng or 1 µL of 0.1 mg/mL solution) and incubated at room temperature with agitation for 1 h. Previously blocked streptavidin agarose beads (blocked with 5 g/L BSA in TBS) were used as an affinity resin to capture the biotinylated Fc, along with phage clones displaying Fc-binding peptides. Phage clones were eluted with 1 mL of 0.2 M glycine pH 2.4 (0.2 M Glycine adjusted to pH 2.4 with HCl). The supernatant was transferred to a separate tube after centrifugation, and neutralized with 150 µL of 1.5 M Tris pH 8.8. Eluted phages were amplified as described in the Ph.D.-7 phage-peptide library manual. 

The number of phage particles in the second and third panning rounds were reduced to 2 × 10^9^ for each round to increase the stringency of selection. The Tween 20 concentration was increased to 0.5% for all binding and washing steps in the second and third rounds to prevent non-specific binding. In order to further ensure that we did not select clones due to non-specific binding, negative streptavidin bead selection was performed in the second and third panning cycles. Prior to incubation with the Fc target, the phage particles were incubated with blocked streptavidin beads for 30 min after which the tubes were centrifuged. The supernatant used for panning with Fc, and streptavidin beads with non-Fc binders were discarded. In this way, all components of the panning system were controlled to ensure only Fc binders will be present after three panning rounds. 

Round 2 clones were eluted twice, first with 2 µM protein A (possibly a direct Fc-binding competitor depending on where on the Fc peptides bind) and then with 0.2 M glycine, pH 2.4. Round 3 was performed similarly to Round 2 with two simultaneous panning experiments. Phage particles for each panning experiment were previously eluted either using protein A or glycine. After each panning experiment phage particles were again eluted by protein A followed by glycine for a total of 4 distinct phage pools at the end of Round 3. Phage pools were titered and plaques picked for confirmation of Fc binding by ELISA. 

### 2.4. ELISA Binding Experiments

#### 2.4.1. Initial Clone Screening

A total of 44 clones were picked from the 4 enriched phage pools for Fc binding confirmation and further analysis by ELISA. The first ELISA plates were intended to demonstrate which clones were positive hIgG4 binders; further selecting against weak binders or those binding something other than hIgG4. Odd rows (A, C, E, and G) of clear, flat-bottomed 96-well polystyrene plates were coated with 200 µL of 100 µg/mL hIgG4 in TBS for at least 24 h at 4 °C with agitation. Even rows (B, D, F, and H) served as negative control rows for each clone as they were filled with TBS only. Plates were blocked with 5 g/L BSA overnight at 4 °C with agitation prior to use. Clones were screened by adding 2.5 × 10^11^ phage in 100 µL in two wells, one coated with hIgG4 and the other blank and serially diluting each five-fold for two more wells, giving six wells total for each clone. In this way, 15 clones could be screened per plate, leaving six wells as the plate background. 

Phage dilutions in TBS with 0.5% Tween 20 (TBST) were performed on a separate blocked 96-well plate to prevent adsorption during dilution. 2.5 × 10^11^ phage particles in 100 µL is equivalent to only a 4 nM phage concentration; therefore lower binding energy clones would not give a positive signal. An anti-M13 phage HRP-conjugated antibody and ABTS HRP substrate were used as the phage detection system. Criteria for positive clones included a signal of at least 0.3 A.U. and signals from hIgG4 coated wells were at least three times higher than the corresponding signal from the negative control uncoated wells. Plates were read at 405 nm (1 s per well) on a Victor X5 multimode plate reader from Perkin Elmer (Waltham, MA, USA) 40 min after ABTS was added to plates.

#### 2.4.2. Peptide-Phage Binding in Cell Culture Conditions

Since these peptides could be used as antibody purification ligands positive clones from the initial ELISA assays were subject to more rigorous testing to confirm binding in conditions resembling cell culture media with other proteins present. Each positive clone from the previous assay (Section 2.4.1) was tested further by exploring binding to hIgG4 in DMEM with 5% FCS and 5 g/L ovalbumin. Plates were coated as in the previous Section 2.4.1 and clones were assayed in triplicate (2.5 × 10^11^ phage particles in 100 µL per well). Instead of no coating, control wells were coated with 200 µL of 100 µg/mL lactoferrin in TBS, again to control for Fc specificity. The original phage library was assayed as well (coated and uncoated wells).

#### 2.4.3. Peptide–Phage Binding to Different Antibody Isotypes

All previous binding assays used the same hIgG4 Fc or full antibody as the target. Peptide binding to multiple antibody types was also investigated. Plates were coated with seven other types of antibodies—hIgG1, hIgG2, hIgG3, hIgM, canine IgG, murine IgG, and rat IgG. Plates were coated with 200 µL of 100 µg/mL for each antibody with the exception of hIgG1 which was at 20 µg/mL. 2.5 × 10^11^ phage particles per well were assayed.

#### 2.4.4. Competitive Binding ELISA with Protein A

A separate plate was again coated with 200 µL of 100 µg/mL hIgG4. ELISA conditions were identical to those in Section 2.4.1 except for the addition of 0.2 µM native protein A to one virus-peptide solution (the other did not have protein A present) for each individual peptide clone prior to addition to coated wells.

### 2.5. DNA Sequencing of Positive Clones

DNA from positive Fc-binding clones was purified and sequenced to determine peptide composition. DNA was selectively isolated from viral protein via sodium iodide precipitation. DNA was sequenced on an ABI 3130 xL Genetic Analyzer (Life Technologies Corp., Carlsbad, CA, USA) prepared with ABI BigDye Terminator v3.1 sequencing chemistry at the Colorado State University Proteomics and Metabolomics Facility. The sequencing primer used was a downstream (−96 nt) complimentary strand primer with sequence 5′ CCCTCATAGTTAGCGTAACG 3′ as suggested in the manual. Therefore sequencing data revealed the anticodon sequence.

### 2.6. Computational Methods

#### 2.6.1. Molecular Docking

**Figure 1:**
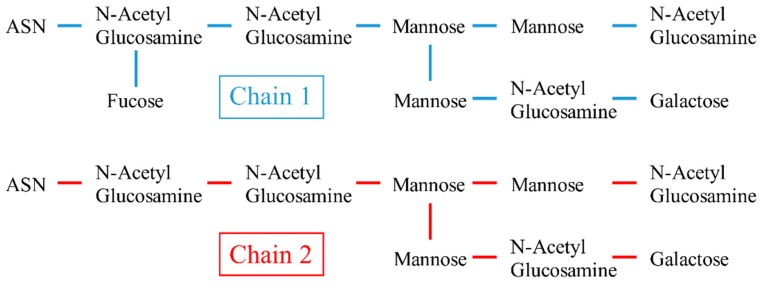
The structures of two glycan chains in the hIgG4 Fc fragment. Glycan chain 1 and 2 connect to the Fc fragment of hIgG4 by the *N*-glycan linkages on two ASN residues.

The molecular docking of the seven experimentally selected heptapetides on the Fc fragment was conducted using AutoDockTools (ADT) 1.5.6 and AutoDock 4.2.6 [56]. ADT initially searched possible conformations of ligands to find the best complementary positions on the surface of the Fc fragment and then determined a global minimum for each ligand using a score function. The hIgG4 Fc fragment used in the docking studies was obtained from the X-ray crystallography RCSB PDB (PDB: 4C55) [57]. The Fc fragment consists of two heavy chains. Chain A consists of 208 amino acid residues and nine carbohydrate residues (Glycan chain 1). Chain B consists of 206 amino acid residues and eight carbohydrate residues (Glycan chain 2). Glycan chain 1 and 2 are connected to the Fc fragment by the N-glycan linkages on two ASN residues as shown in Figure 1. The atomic structures of seven heptapeptide ligands were constructed using AMBERTOOLS package [58]. 

Before importing the ligands and the hIgG4 Fc fragment in ADT, all hydrogen atoms were removed. ADT automatically added polar hydrogen atoms to the complex to evaluate the formation of hydrogen bonds which were the most important interactions in molecular docking. The hydrogen atoms of the water molecules in the Fc fragment crystal structure were added by Chimera [59]. The Lamarckian Genetic Algorithm [48] method was used to evaluate the geometry complementarity. In this procedure, the pose of a ligand was encoded into linear chromosomes including translational and rotational information. Each chromosome contains information on coordinates, movements, and reference structures that relate to the conformational change of ligands [60]. Two-step docking processes were conducted to obtain more accurate results. The first step uses flexible ligands and the rigid receptor to find key residues on the receptor. The second step allows both key residues on the receptor and the ligands to be flexible. The complex structure with the strongest binding affinity was chosen as the initial coordinates for the MD simulation of the Fc-ligand complex.

#### 2.6.2. Molecular Simulations 

MD simulations were conducted using AMBER [61]. The state of titratable amino acids was evaluated by AMBERTOOLS and validated by APBS [62,63,64] at pH 7 with implicit solvent model. The ff14SB force field was used for the amino acids. This force field was developed based on the original ff99SB force field developed by the Simmerling’s group [65]. The current version of the force field has a better energy balance between side chains and backbones for proteins. The GLY backbone and β-turn structures also behave better. GLYCAM_06j force field [66] was used for the glycan chains on the Fc fragment. The carbohydrate force field has better compatibility with ff14SB. 

The truncated octahedral unit cell was used. The edge length is about 37 Å. Each unit cell contains one Fc fragment, one ligand and 17,400 water molecules. The number of atoms per unit cell is around 59,000. The TIP3P water model [67] was used. TIP3P water model has been successfully applied to previous studies [68,69,70]. The buffer distance between the complex and the box edge was 10 Å. All simulations were run at a constant temperature (300 K) and pressure (1 bar) (NPT) under the periodic boundary condition with the Langevin dynamics thermostat [71] to improve conformational sampling characteristics. A 10 Å cutoff was used for the real part of the electrostatic and for the van der Waals interactions. The time step was chosen to be 1 femtosecond (fs). A hydrogen bond (H-bond) is formed if the distance between the two heavy atoms A and B is less than 3.5 Å and the angle A—H···B is greater than 150°. 

## 3. Results

### 3.1. Phage Display Panning Results

In Round 1 of panning, phage particles were eluted from Fc bound to streptavidin beads via a biotin linker with a strongly acidic glycine buffer. This resulted in a phage titer of 6.7 × 10^5^ pfu/mL. This relatively high titer after only one round of enrichment for specific Fc binders most likely indicated numerous non-specific interactions of phage-peptides with other species present such as the streptavidin agarose beads. Negative selection with streptavidin beads and amplified Round 1 phage eluate (2 × 10^9^ pfu input) prior to panning with the Fc target was successful in reducing non-specific binding of phage particles as shown by the lower titers from both protein A (5.0 × 10^4^ pfu/mL) and subsequent acidic glycine (2.8 × 10^5^ pfu/mL) eluate pools. This also demonstrates the level of non-specifically interacting peptide-phage in the original library that need to be removed from the panning pool before successful Fc binders can be isolated. 

Before switching to a solution phase affinity bead panning system several attempts were made to isolate Fc binding peptides with Fc coated to a polystyrene plate. High final round panning eluate titers (greater than 1 × 10^9^ pfu/mL) were observed and consensus sequences were reached however all ELISA binding assays were either negative or showed similar binding to negative control (uncoated) wells as the positive wells indicating polystyrene or, less probably, BSA binding. However, the final round of unamplified eluate titers from the solution panning was in the 10^5^–10^6^ pfu/mL range. This, combined with the small amount of target Fc present (2 pmol) and input phage particles (less than 2 × 10^9^ pfu/mL, after negative selection) during solution panning, gave higher confidence that specific peptide-Fc interactions were dominant. 

### 3.2. Confirmation of Selected Clone Binding by ELISA

Previously, non-specific clones had been selected from surface panning experiments; therefore, initial binding characterization of selected clones was essential before more detailed binding experiments. As stated in Section 2.4.1, 44 total clones were isolated from titer plates and amplified for characterization by ELISA. Of those 44, 15 were from plates eluted with acidic glycine in the second panning cycle and 2 µM protein A in the third panning cycle. Another 15 were from plates eluted with protein A in both the second and third panning cycles. An additional 14 clones were from plates eluted with acidic glycine buffer in both the second and third panning cycles.

Two criteria were used for selecting positive clones. The signal in the hIgG4 coated well with the most phage (2.5 × 10^11^ particles) had to be at least 0.3 A.U. At such a low concentration (4 nM in 100 µL) of phage particles a positive signal was only possible if the dissociation constant of the peptides was low (less than µM), increasing the selection of peptides with high binding energy. The second criterion was the ratio of the signal from hIgG4 coated wells and uncoated wells (non-specific control) had to be greater than 3. Again, this provided assurance that selected clones were binding to the Fc portion of the antibody and not unblocked portions of the polystyrene well.

**Figure 2:**
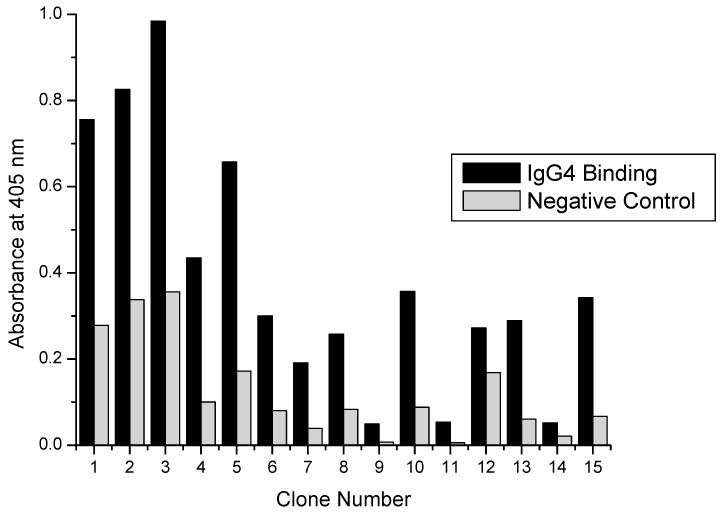
ELISA results showing hIgG4 binding by peptide-virus clones eluted with (second round) 5 µM Protein A followed by (third round) 0.2 M glycine pH 2.4.

**Figure 3:**
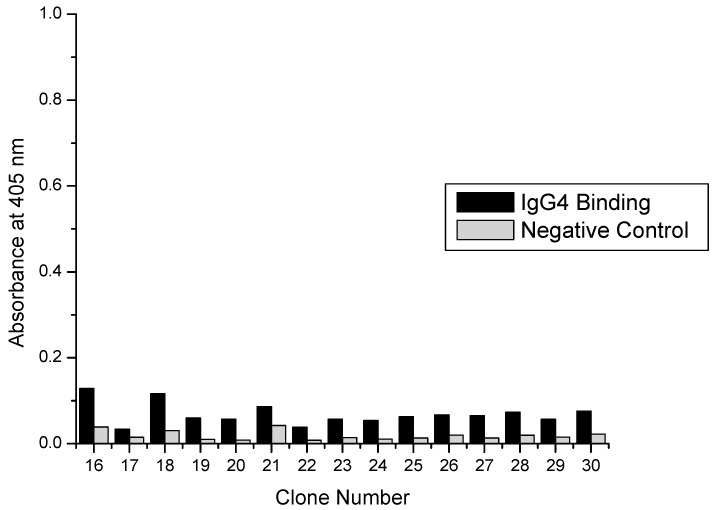
ELISA results showing hIgG4 binding by peptide-virus clones eluted with (second round) 5 µM Protein A followed by (third round) Protein A.

**Figure 4:**
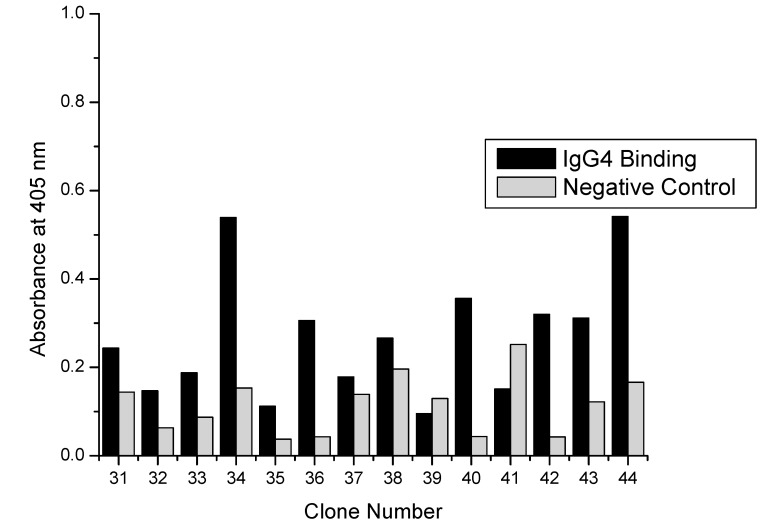
ELISA results showing hIgG4 binding by peptide-virus clones eluted with (second round) 0.2 M glycine pH 2.4 followed by (third round) 0.2 M glycine pH 2.4.

Figure 2 shows the binding results for clones eluted with acidic glycine buffer and protein A. Elution conditions for round two and three panning were acidic glycine and protein A, respectively. Three clones met the selection criteria and further binding studies included these clones. Figure 3 shows binding results from clones eluted with protein A (second round) and protein A again (third round). Interestingly none of the clones isolated from these elution conditions showed strong enough binding to evaluate further. Figure 4 presents the binding results from the acidic elution conditions. Clones 31–44 were isolated using acid elution conditions in both the second and third rounds. Four clones satisfied the selection criteria and were further evaluated as well. 

### 3.3. Secondary Screening of Clones

**Figure 5:**
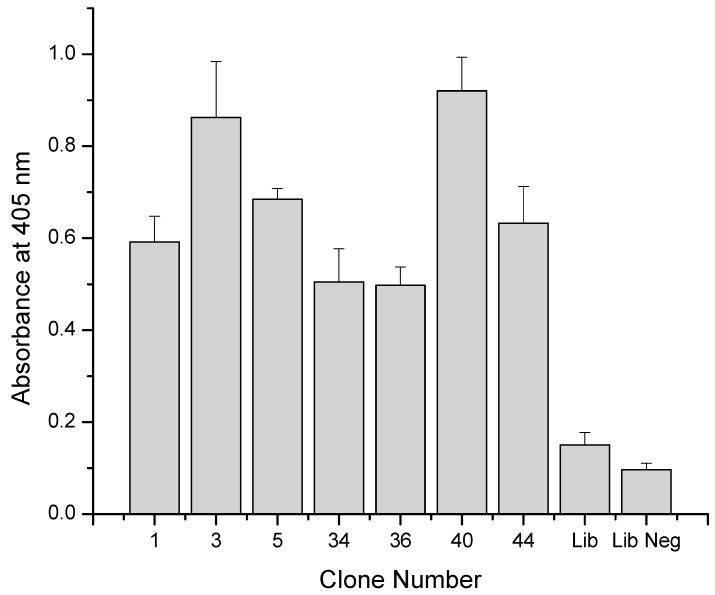
ELISA results for peptide binding to hIgG4 in DMEM, 5% fetal calf serum, and 5 g/L BSA. Original phage library (Lib) binding to hIgG4 coated wells and original library binding to uncoated wells (Lib Neg) are presented as well.

Seven total clones were screened further to confirm selective binding to hIgG4. The binding conditions in this round of ELISA were designed to mimic antibody binding from cell supernatant. Therefore, binding to hIgG4 was attempted in DMEM cell culture media with 5% FCS and 5 g/L ovalbumin (BSA was present as a blocking agent as well). The presence of ovalbumin at orders of magnitude higher concentration compared to the peptide-virus clones, as well as the proteins and other molecules associated with FCS established a challenging binding environment for the peptides to hIgG4. Figure 5 shows the secondary selection results. Similar to the uncoated negative control well results during the screening ELISA, all seven clones showed at least three-fold greater absorbance in hIgG4-coated wells than in lactoferrin-coated negative control wells (data not shown). All clones also showed greater than 2.5-fold higher absorbance on IgG4-coated wells than the original phage library indicating successful enrichment of IgG4-binding clones. Binding signal of the original phage library to IgG4-coated wells was not statistically different from the phage library signal to negative control wells indicating very low positive binding as expected in the non-enriched library. Clones 3 and 40 showed the highest binding with ELISA signals above 0.85 AU, representing over four-fold enrichments in ELISA signal as compared to the original library. As indicated by the error bars which represent the standard deviation, the results were highly reproducible.

### 3.4. Binding to Multiple Antibody Types

**Figure 6:**
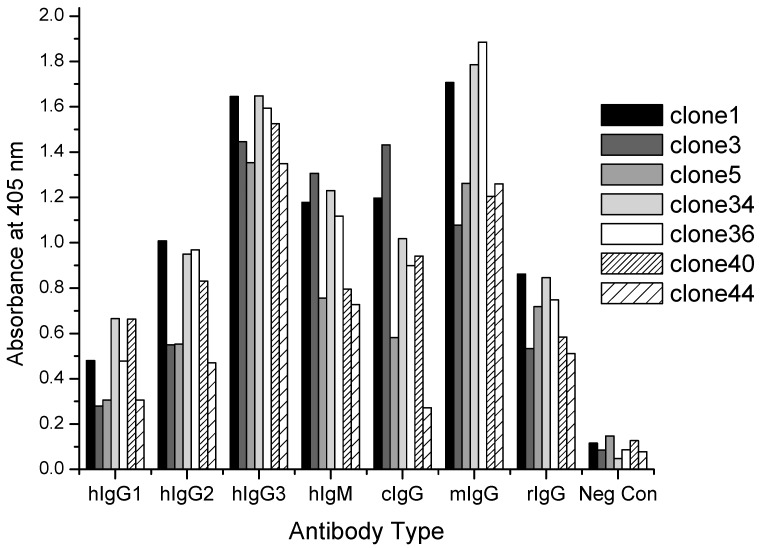
Peptide binding to various human antibody isotypes, as well as canine IgG (cIgG), murine IgG (mIgG), and rat IgG (rIgG).

To further characterize the binding of the peptides to antibody Fc ELISA plates were coated with multiple antibody types. Native Protein A has strong affinity for human IgG1, IgG2, and IgG4 antibody isotypes but not IgG3. Therefore peptide binding to all 4 different human IgG isotypes was tested as well as binding to human IgM which has shown variable binding to protein A [72]. Binding to non-human IgG molecules such as murine IgG, canine IgG, and rat IgG was also investigated. The ELISA binding results from the multiple antibody plate are shown in Figure 6. With the exception of binding to human IgG1, all peptides demonstrated strong binding to all antibody types tested. It was difficult to conclude the peptides do not bind to hIgG1 because the well coating concentration was five-fold lower than the other antibodies as a result of limited available hIgG1 reagent. Higher ELISA signal was observed for all peptides binding to hIgG1 as compared to uncoated negative control wells; however, the signal was not at least 3X higher to match the selection criteria set forth previously. Interestingly, clone 44 showed strong binding to all antibody types with the exception of binding to canine IgG (and hIgG1, previously explained). In general, the peptides bound strongest to both murine IgG and hIgG3, which is particularly interesting given the lack of hIgG3 affinity for protein A.

### 3.5. Competitive ELISA with Protein A

To determine whether selected peptides shared portions of the same binding site as protein A, a competitive binding ELISA was conducted. The results of the competitive ELISA are not presented in this contribution because the assay as intended was a failure. However, an interesting outcome of the ELISA was all wells wherein protein A was added showed at least 2× higher signal than non-protein A wells, which is the opposite of what was expected of a competitive binding assay. On the surface, these data are hard to reconcile until the nature of the protein A binding protein is considered. Multiple studies have characterized the protein A binding stoichiometry as greater than 2.4 to 1 referring to the ratio of antibody molecules to protein A molecules [73,74]. When considering the multi-valency of the protein A ligand, it is hypothesized that protein A is binding to the immobilized hIgG4 and is subsequently sandwiched by a second antibody which is the HRP-conjugated reporter antibody present in the ELISA assay. Therefore, high ELISA signal was considered independent of bound peptide-viral particle concentration when Protein A was present.

### 3.6. Sequencing of Selected Peptides

Peptides displayed on selected phage clones were sequenced at the Colorado State University Proteomics and Metabolomics Facility. Sequencing results are presented in Table 1. All seven selected peptides were single heptapeptide displays; previous panning attempts (less stringent) had resulted in a high percentage of multi-heptapeptide constructs which were subsequently shown to bind in a non-specific manner. The unique heptapeptides selected in this work bound specifically to the Fc region of multiple antibody types.

### 3.7. Computational Results

#### 3.7.1. Molecular Docking to Determine Binding Site and Binding Energy

**Figure 7:**
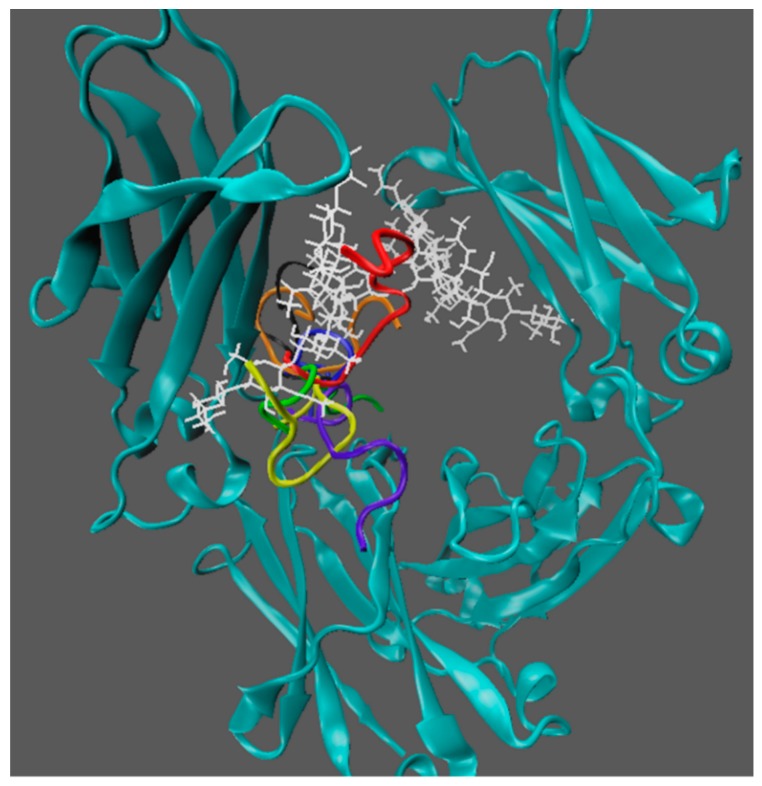
The binding locations of seven heptapeptide ligands in the Fc fragment from the molecular docking studies. Ligand 1–7 are colored blue, red, orange, green, black, yellow, and violet respectively. Glycan chains are shown in white.

CBS binding site was considered previously to be the only binding site on the Fc fragment. Some previous MD simulations also found the CBS region to be the binding site for small ligands. However, only part of the Fc fragment was included in those studies. In order to elucidate the binding mechanism(s) of the seven heptapeptides with the Fc fragment, docking scanning of the entire Fc fragment (two heavy chains and two glycan chains) surface has been conducted. As discussed, the study was initially conducted by the rigid docking followed by the flexible docking in AutoDock. During this simulation section, for the sake of convenience, clones 1, 3, 5, 34, 36, 40, and 44 are renamed ligand 1–7 respectively. The poses of the ligands and binding sites associated with the lowest binding energies for the seven heptapeptides were shown in Figure 7. It can be seen that the binding sites for all the seven ligands are located inside the Fc fragment close to the glycan chains. 

The binding free energies between the seven ligands and the Fc fragment from the docking studies are tabulated in Table 2. The results indicated that ligand 2 (clone 3) has the most favorable binding interaction with the Fc fragment with the lowest binding free energy of −6.36 kcal/mol. Ligand 6 (clone 40) has the second lowest binding energy with Fc. Ligands 4 (clone 34) and 5 (clone 36) have the least favorable binding free energies. These results are in excellent agreement with the experimental data as shown in Figure 5. 

#### 3.7.2. Atomistic MD Simulations

**Figure 8:**
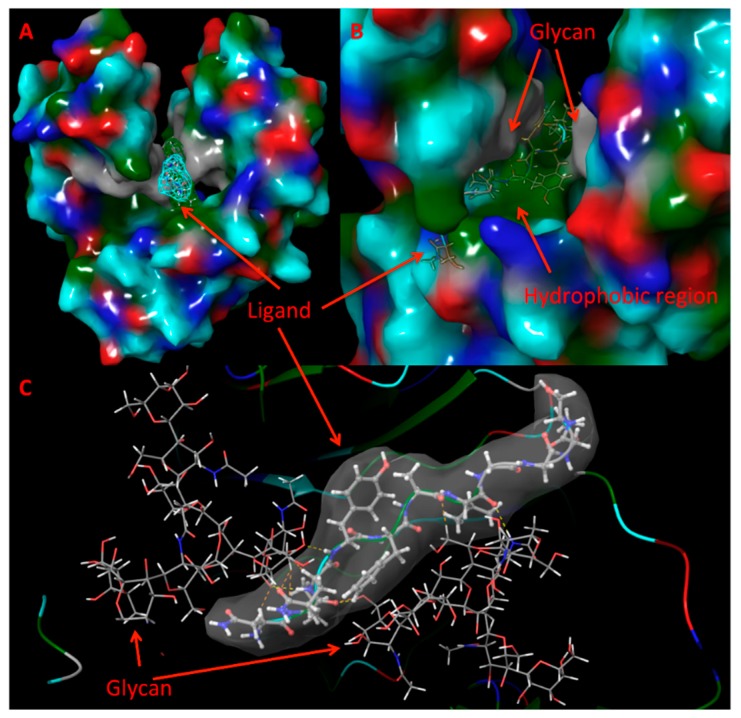
Interaction between ligand 2 and the Fc fragment. Panel A shows the binding pocket. The surface of the Fc fragment shown: GLY and glycan chains (gray), hydrophobic residues (dark green), polar uncharged residues (cyan); positively charged residues (blue), negatively charged residues (red). Ligand 2 is shown by a mesh surface. Panel B is the enlarged view of the binding site. Panel C exhibits hydrogen bonding (dashed lines) interaction between glycan chains and ligand 2. Gray is the hydrophobic residues.

To illustrate the binding mechanism(s) more clearly, ligand 2 (clone 3) which has the strongest binding with the Fc region was chosen to conduct atomistic MD simulations together with the Fc fragment in aqueous solution for a total of 150 ns. The initial structure of ligand-Fc complex was from the docking studies. The location of ligand 2 inside the Fc region was shown in Figure 8 from the MD simulations. Ligand 2 was found to bind deep in the Fc fragment as shown in Figure 8A. It is located between the two glycan chains and above the hydrophobic belt as shown in Figure 8B. Both glycan chains form hydrogen bonds with ligand 2 as shown in Figure 8C. The cavity where ligand 2 binds is formed by the two glycan chains and the hydrophobic residues on the Fc region. This cavity deep in the Fc fragment is suitable for small ligands. The binding pocket is a binding site for ligands that tend to form strong hydrogen bonding interaction with the glycan chains and the hydrophobic interaction with the hydrophobic amino acids. Similar MD simulations were also conducted for the other ligands. All seven heptapeptides showed binding at the same pocket indicating a similar mechanism. 

**Figure 9:**
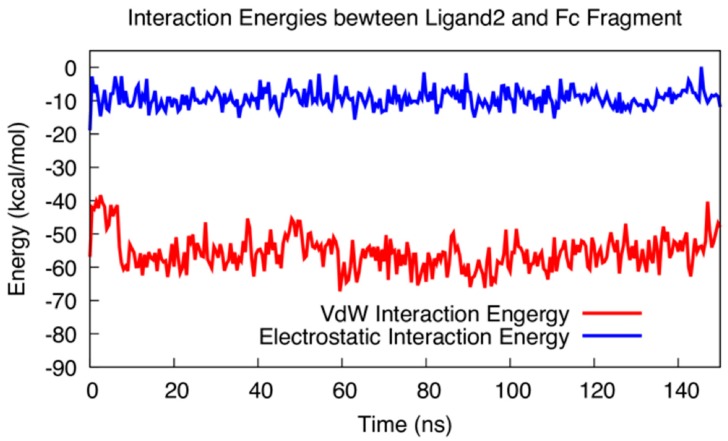
VdW and electrostatic interaction energies between ligand 2 and the Fc fragment during the 150 ns simulation period.

Figure 9 shows the interaction energies between ligand 2 and the Fc fragment during the 150 ns simulation period. The interaction energy was divided into two terms, van der Waals (VdW) and electrostatic interaction energies. A dielectric constant of 4 was used to determine the electrostatic interaction energy. Earlier studies [75,76] indicated that the dielectric constant is different for different materials and a value of 4 [77] is appropriate for protein interactions in aqueous solution. It can be seen that electrostatic interaction energies (~−10 kcal/mol) were less negative than VdW interaction energy (~−55 kcal/mol). This indicates that the binding is dominated by VdW interaction, and maximized VdW contact is critically important in Fc–ligand interactions. Both electrostatic and VdW interaction energies have only small fluctuations indicating the stability of the binding complex formed. 

**Figure 10:**
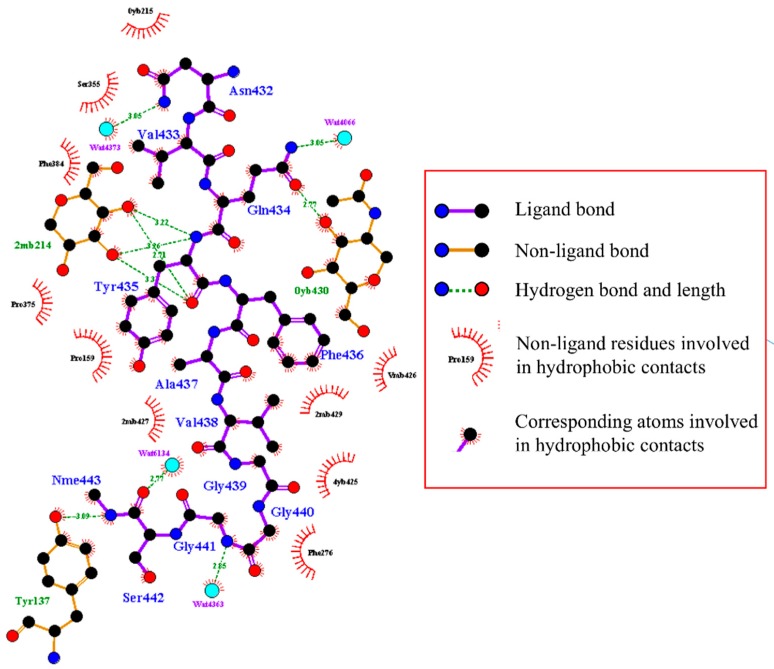
2D diagram of interactions between ligand 2 and the surrounding residues at the Fc fragment. Structure water molecules are also selected. Ligand 2 is presented by sticks and balls. Water molecule is cyan ball.

In order to elucidate the specific binding interactions involved between the Fc fragment and ligand 2, a snapshot of molecular interactions between the ligand and the surrounding residues of the Fc fragment was taken at 100 ns simulation time when the stable complex has been persistently formed. A 2D molecular interaction diagram calculated by LigPlot+ [78,79] was shown in Figure 10. The diagram portrays the hydrogen bonding interaction and hydrophobic contacts between ligand 2 and the Fc fragment. 

It can be seen from Figure 10 that ligand 2 interacts with amino acids (PHE276, SER355, PHE384, PRO375, PRO159) and carbohydrate residues (0YB215, 2MB427, 2MB429, VMB426, 4YB425) on the Fc fragment in this binding site. It is noticeable that most parts of ligand 2 are not solvent accessible. A few structured water molecules (WAT4066, WAT4373, WAT6134, WAT4363) were also involved in the hydrogen bonding interaction with the ligand. Moreover, it can be found that GLN434, TYR435, NME443 on ligand 2 can form hydrogen bonds with residues on the Fc fragment. ASN432, GLN434, GLY441, and NME443 form hydrogen bonds with structured water molecules trapped in the binding cavity. 

**Figure 11:**
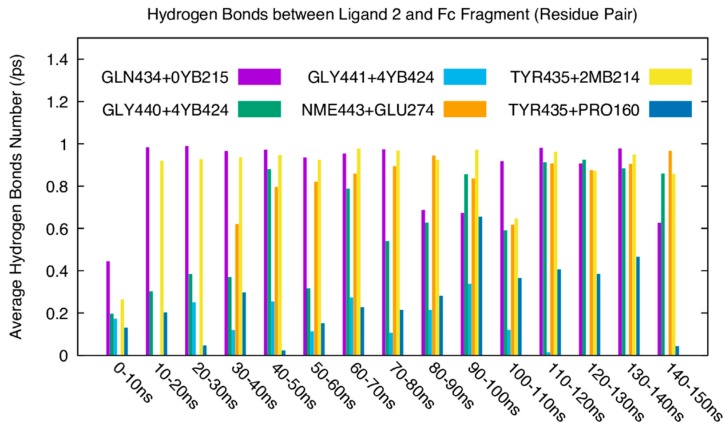
Average number of hydrogen bonds formed between ligand 2 and the Fc fragment in each 10 ns during the whole 150 ns simulation.

Figure 11 shows the average number of hydrogen bonds formed between residues on ligand 2 and the groups on the Fc fragment at each 10 ns during a total of 150 ns simulations. For each pair of hydrogen bonding interaction, the first ones come from the ligand (GLN434, GLY441, TYR435, GLY440, NME443), the second ones come from the glycan on Fc fragment (0YB215, 4YB424, 2MB214). It is found that GLN434–0YB215, GLY441–4YB424, TYR435–2MB214, and GLY440–4YB424 form persistent hydrogen bonding interactions. Although the number of hydrogen bonds formed between the residue pairs varies from time period to time period, the average number of hydrogen bonds for the pairs is close to 1. This indicates that glycan chains clearly dominate the hydrogen bonding interaction between ligand 2 and the Fc fragment. 

## 4. Discussion

This study was successful at identifying seven unique heptapeptides with specific affinity for the Fc region of immunoglobulins. The importance of the phage display selection system and multiple screening and selection rounds were highlighted for effective peptide selection. Identified peptides were shown to have broader binding tropisms than protein A as evidenced by peptide binding to all major human IgG isotypes including IgG3, human IgM, and canine, murine, and rat IgG. Binding was shown to not be inhibited by the presence of fetal calf serum, bovine serum albumin, ovalbumin, or DMEM cell culture medium. 

It was interesting that, even after stringent efforts put forth to remove non-specific clones, only 20.5% of the clones were positive Fc and hIgG4 binders. Though there is the possibility that, because panning was performed with Fc only whereas the ELISA’s were all performed with intact antibody, several peptides initially selected may have bound Fc near the N-terminal hinge region normally connected to the Fab portion of the antibody. That would preclude peptide binding of the intact antibody, which was the goal of this study. The stringent selection conditions—three rounds of phage display panning, as well as the relatively large Fc binding surface—may account for the lack of overall consensus sequence obtained in this study. The screening ELISA and secondary selection ELISA data showed positive clones were present after three rounds of panning and therefore subsequent panning rounds were not undertaken. Four or more rounds of panning may have led to a consensus sequence, however further rounds of panning can lead to selection not on the basis of binding but selection by viral reproductive characteristics.

Though each peptide identified was unique they did exhibit some common characteristics. Histidine residues were present in five of the seven peptides selected; serine and lysine residues were also present in five of the seven peptides. Histidine, and to a lesser extent lysine and serine, are common residues in several previously identified Fc binding peptides such as CHKRSFWADNC from Jeong et al., or the HWRGWV peptide first identified by Yang et al. [19,22]. Histidine and lysine residues are both highly conserved in the five homologous Fc binding domains present in native protein A (NCBI GenBank). Protein BLAST analysis on the NCBI website showed each selected peptide sequence is present in a multitude of different functional and structural proteins, however none contain known immunoglobulin binding domains providing further evidence these peptides are novel. Molecular docking and MD simulations were conducted to elucidate the binding mechanisms and energetics for the seven heptapeptides to the Fc region. It was found that all seven peptides bind to the cavity located between the glycan chains. Binding free energies and the order of binding strength obtained from the docking studies are consistent with the ELISA experimental data. MD simulations further elucidate the interactions between the strongest binding peptide (clone 3, ligand 2) with Fc region. Both hydrogen bonding and hydrophobic interactions play a dominant role in the binding mechanism. 

A library of heptapeptides was chosen to evaluate for Fc binding for several reasons. No heptapeptides specifically binding Fc have been reported in the existing public literature. Existing available heptapeptide libraries enabled relatively quick exploration without the need for library generation. Importantly, these short heptapeptides, as well as other Fc-binding peptides, may show greater chemical stability than native or recombinant protein A ligands. Their short primary sequence, in this case seven amino acids total, eliminates the need to preserve any tertiary or secondary structures present in larger binding ligands. This may make peptides less susceptible to denaturing or chemical alteration in sanitizing conditions such as 1 M NaOH, while current protein A ligands are highly sensitive to such conditions. Finally, these peptides may prove useful in the purification of Fc-containing immunoglobulins, such as monoclonal antibodies, from serum or cell culture supernatant.

Identification of multiple peptides which specifically bind to a given target (in this case Fc) may be beneficial to engineering ligands with higher specificity. Several peptides or small binding domains joined together have been shown both in natural and engineered ligands to increase specificity [80,81] including protein A itself [82].

## 5. Conclusions

A stringent solution panning phage display method has been used to identify seven unique heptapeptides that have specific affinity for the Fc region of immunoglobulins. Applying multiple ELISA screening and selection rounds confirmed selective binding of the peptides to Fc. Binding of the peptides to human IgG1, IgG2, IgG3, IgG4, and IgM (protein A does not bind hIgG3) was demonstrated as well as binding to canine, murine, and rat IgG. Due to selective binding for Fc and ease of production and use, these peptides may be useful in antibody detection and purification applications.

## Figures and Tables

**Table 1:** Sequencing results from clones showing positive Fc binding.

Clone	Amino Acid Sequence
1	Cys-Pro-Ser-Thr-His-Trp-Lys (CPSTHWK)
3	Asn-Val-Gln-Tyr-Phe-Ala-Val (NVQYFAV)
5	Ala-Ser-His-Thr-Gln-Lys-Ser (ASHTQKS)
34	Gln-Pro-Gln-Met-Ser-His-Met (QPQMSHM)
36	Thr-Asn-Ile-Glu-Ser-Leu-Lys (TNIESLK)
40	Asn-Cys-His-Lys-Cys-Trp-Asn (NCHKCWN)
44	Ser-His-Leu-Ser-Lys-Asn-Phe (SHLSKNF)

**Table 2:** Relative binding energies of seven ligands estimated by molecular docking.

Clone	Ligand	ΔΔ*G*_binding_ (kcal/mol)
1	1	−4.47
3	2	−6.36
5	3	−3.51
34	4	−2.22
36	5	−2.67
40	6	−4.67
44	7	−3.11
